# Recovery in Supported Accommodations: A Scoping Review and Synthesis of Interventions for People with Severe Mental Illness

**DOI:** 10.1007/s10597-020-00561-3

**Published:** 2020-02-03

**Authors:** Neis Bitter, Diana Roeg, Chijs van Nieuwenhuizen, Jaap van Weeghel

**Affiliations:** 1 Department of Social and Behavioural Sciences, Tranzo Scientific Center for Care and Wellbeing, Tilburg University, PO Box 90153, 5000 LE Tilburg, The Netherlands; 2 GGzE Institute for Mental Health Care, PO Box 909, 5600 AX Eindhoven, The Netherlands; 3 Phrenos Centre of Expertise, PO Box 1203, 3500 BE Utrecht, The Netherlands; 4 Parnassia Group, Dijk en Duin Mental Health Centre, PO Box 305, 1900 AH Castricum, The Netherlands

**Keywords:** Mental health recovery, Societal participation, Severe mental illness, Supported accommodation, Supported housing

## Abstract

Research on the recovery domains beside clinical recovery of people with severe mental illness in need of supported accommodations is limited. The aim of this study was (1) to investigate which recovery interventions exist for this group of people and (2) to explore the scientific evidence. We conducted a scoping review, including studies with different designs, evaluating the effectiveness the recovery interventions available. The search resulted in 53 eligible articles of which 22 focused on societal recovery, six on personal recovery, five on functional recovery, 13 on lifestyle-interventions, and seven on creative and spiritual interventions. About a quarter of these interventions showed added value and half of them initial promising results. The research in this area is still limited, but a number of recovery promoting interventions on other areas than clinical recovery have been developed and evaluated. Further innovation and research to strengthen and repeat the evidence are needed.

## Introduction

Most people with severe mental health problems can recover and live in the community with or without support (Keet et al. 2019). A relatively small group of people (10–20%) has long-term, severe and complex needs but consumes 25–50% of the mental health and social care budget (Killaspy et al. 2016). Killaspy et al. (2016) therefore referred to this group as a ‘low volume, high needs’ group. These people often have major negative and ongoing positive symptoms in addition to other mental, social and physical health problems. They need the permanent support of supported housing facilities or residential care (Killaspy 2016; Leff et al. 2015; Sandhu et al. 2017; van Hoof et al. 2015). These services offer practical daily care, nursing and support to persons with severe mental illness (SMI) in their daily lives, aiming at improvements in recovery and functioning. Nevertheless, people with long-term SMI still report unmet needs concerning health, work, social relations and daily activities (Bitter et al. 2016; de Heer-Wunderink et al. 2012a, b).

Over the past two decades, there have been increasing attention for what it means to recover from a mental illness. There is a growing recognition that recovery is more than the remission of psychiatric symptoms. The current vision is that recovery is ‘a way of living a satisfying, hopeful and contributing life even with limitations caused by illness’ (Anthony 1993). Several authors described that recovery comprises multiple aspects (Couwenbergh and van Weeghel 2014; Davidson et al. 2005; Leamy et al. 2011; Resnick et al. 2005). An example of a classification that is used often in the Netherlands is: clinical, functional, social and personal recovery (Couwenbergh and van Weeghel 2014). First, clinical recovery refers to a decrease in clinical symptoms such as hallucinations, anxiety or depressive feelings (Liberman et al. 2002). The other dimensions are of more recent attention. Functional recovery refers to executive functioning such as planning and problem solving (Savla et al. 2012). Societal recovery is about regaining everyday functioning in areas such as work, social relationships, housing and leisure (Farkas and Anthony 2010). Personal recovery refers to a person’s own experience and is about hope, empowerment, self-determination and regaining the identity of someone who is living a meaningful life despite the presence of symptoms (Anthony 1993; van Gestel-Timmermans et al. 2012a). Recovery dimensions are closely related and influence each other constantly in complex processes (Davidson et al. 2005).

Treatment and support for people with SMI therefore should ideally focus on all dimensions of recovery and be tailored to a person’s individual needs (Bitter et al. 2016; van Weeghel et al. 2019a). Several types of psychosocial interventions have been developed to support people with SMI in their recovery on the dimensions next to the clinical one (Slade et al. 2014). Rehabilitation methods, for example, focus on clients’ personal goals and wishes regarding daily life and societal recovery. Examples of well-known methods in this field are the ‘choose-get-keep’ approach, also referred to as Boston psychiatric rehabilitation, (Anthony et al. 2002), illness management and recovery (IMR) (Mueser et al. 2006) and the strengths model of case management (Rapp and Goscha 2006). Other methods focus on a specific aspects of life. These include individual placement and support (IPS) in which people are supported to gain and stay in competitive employment (Burns et al. 2007; Michon et al. 2011). Other methods aim to improve cognitive functioning or practical skills; these include social and independent living skill modules, cognitive remediation programs and cognitive adaptation training (CAT) (Hansen et al. 2012; Marder et al. 1996; Stiekema et al. 2015). More recently, interventions have been developed especially focusing on personal recovery, sometimes provided by experts-by-experience (Boevink et al. 2016; Fox and Horan 2016; van Gestel-Timmermans et al. 2012b).

There is an increasing amount of research on the effectiveness of interventions addressing several outcomes. IPS, for example, has shown to have a strong and consistent effect on vocational outcomes (Michon et al. 2011). Furthermore, the Boston approach has been shown to increase social functioning and goal attainment (Swildens et al. 2011). Studies concerning several other interventions, such as the strengths model and those aimed at personal recovery, have reported varying results (Ibrahim et al. 2014; Lloyd-Evans et al. 2014; Tse et al. 2016).

Although research on these interventions have shown promising results, studies on interventions for clients living in supported accommodations such as residential care and supported housing services, however, lack behind (Chilvers et al. 2006; McPherson et al. 2018). Available studies were executed mainly with participants who live independently with a relative small amount of support. Also, most of the available studies concern interventions that focus on a selective group of motivated clients who can formulate concrete goals (Michon et al. 2011; Swildens et al. 2011). We cannot assume that these practices are suitable and valuable for people with SMI living in supported accommodations, of which is known their needs are more complex and some have lost their motivation and goals in life (Bitter et al. 2016; de Heer-Wunderink 2012).

For that reason, this study aims to identify and evaluate studies on psychosocial interventions focusing on the dimensions of recovery besides the clinical one, in supported accommodation for people with severe mental illness. The findings of this study can contribute to the further development of the content and quality of the support offered by supported accommodation.

### Aims of the Study

With this review, we aim to answer the following questions:

1. Which interventions have been applied and evaluated to support clients with severe mental illness using supported accommodation in their recovery on domains besides clinical recovery?
2. What scientific evidence is available about the outcomes of these interventions?

## Methods

We choose to conduct a scoping review, as these are established for use when the objective is to examine the extent, range and nature of research activity in a certain field and to summarize and disseminate the research findings (Pham et al. 2014). We followed the steps described by Arksey and O’Malley (2005) in their framework for the execution of a scoping review: (a) identify the research question, (b) identify relevant studies, (c) select the studies, (d) chart the data and (e) collate, summarize and report the results.

### Search Strategy

To answer our first research question, we searched the following databases: PubMed, Psycinfo, Embase and Cinahl (January 2018, Update December 2019). These databases were chosen to cover medical (PubMed and Embase) as well as psychological (Psycinfo) and nursing (Cinahl) literature. We formulated and combined search terms concerning: (a) the setting and population (mental disorder/illness, schizophrenia, psychosis, inpatient rehabilitation, supported accommodation, sheltered housing, housing facility, community housing, community facility, supported housing, residential facility and residential care), (b) the scope and outcome of the intervention (psychosocial, societal, recovery, functioning, rehabilitation, health, wellness and cognition), and (c) study type (clinical trial, randomized controlled trial, evaluation study, experimental trial, naturalistic study, follow up study, quasi-experimental and case study).

To select studies that corresponded with our research aims, we formulated inclusion and exclusion criteria. We included peer-reviewed articles that were published in English from January 2000 till December 2019; aimed at adult clients with severe mental illness receiving services from housing services or comparable long-term (> 1 year) supported accommodation; evaluated psychosocial interventions focussing on personal, functional or societal recovery outcomes; evaluated the outcomes of an intervention on the client level; and evaluated outcomes by means of effect evaluation all types of designs except for expert opinions and case studies. As we aimed to give an overview of existing interventions for this group, we also included protocol papers and checked if there were results published already. To be able to provide a clearly defined answer to the research questions and to keep the results manageable, we also formulated exclusion criteria. Studies were excluded if they primarily focussed on substance abuse; intellectual and/or developmental disability, including brain damage; or on homelessness; or if they were executed in developing countries.

### Study Selection Process

In the first and second selection phase, the first two authors each screened a separate part of the titles from the initial search, and of the remaining papers they screened the abstracts on relevance. When there was doubt, the selection was made in consensus. The first and second author determined final inclusion by discussing the interpretation of the inclusion criteria in certain cases. When doubt persisted about an abstract, the article was included so that a more careful decision could be made in the next phase.

In the third phase, the first and second author read the full-text of the remaining articles and made a final selection. In this final phase, both authors each read half of the articles independently. Again, articles about which doubt existed were discussed until consensus was reached. The selected studies then were categorised in a qualitative synthesis, based on the dimensions of recovery: societal, functional and personal, and additional in vivo categories were made when needed.

### Outcome Evaluation

Our second aim was to evaluate what is already known about the outcomes of these interventions. Therefore, the second phase of the qualitative synthesis was evaluation of each study to understand the status of the available evidence of each intervention found. First, we formulated categories of designs based on Evans’ hierarchy of evidence (2003): randomized (controlled) study, uncontrolled longitudinal study, or other (all other designs except case studies and expert opinions). Next, we evaluated the results of relevant outcomes and (where possible) the effect sizes of these results. Again, three options were possible: Large or medium effects, small effects, or neutral, unclear, unknown or not convincing yet. Based on these criteria, we concluded there was one of three options: (a) added value when a randomized control trial (RCT) resulted in small, large or medium effects, (b) promising first results when other designs than RCTs showed positive results, or (c) no evidence for the effectiveness yet when there were neutral or negative results or no results yet. The first and second author executed this quality assessment independently. Each assessed an equal part and then discussed the results until they reached a consensus. This review is part of a larger research project which received ethical approval from the Medical Research Ethics Committee of the Elisabeth Hospital in Tilburg (NL41169.008.12).

## Results

**Fig. 1:**
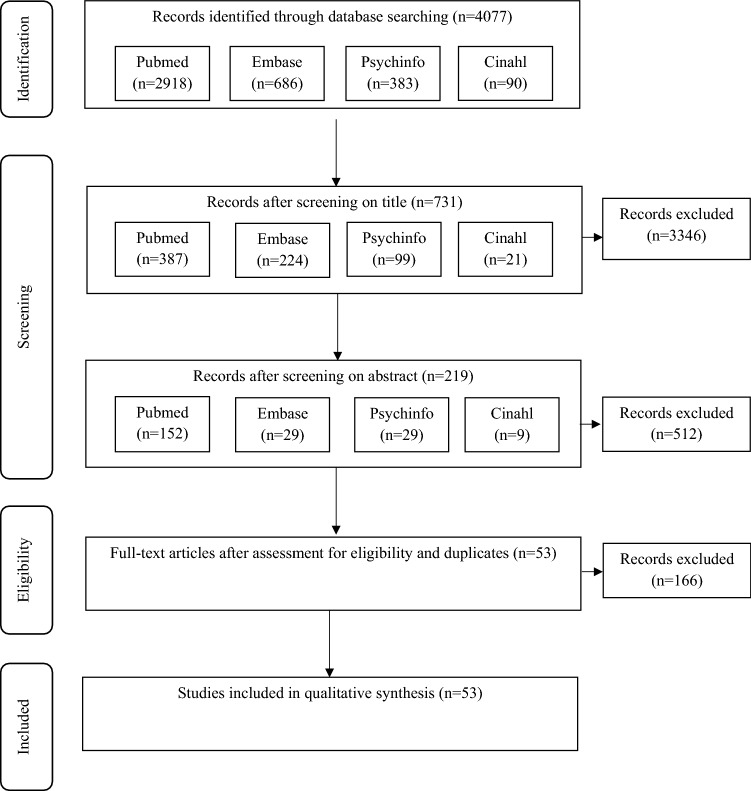
PRISMA flow diagram

Fifty three articles met the inclusion criteria. Figure 1 shows the PRISMA flow diagram of the search, while Table 1 shows the results of the qualitative synthesis of the included articles. Five categories were formed. Three were based on the often distinguished dimensions of the recovery process: societal recovery, personal recovery and functional recovery, and two were formed in vivo: lifestyle, and cultural and spiritual.

**Table 1:** Type, amount and evidence of included studies

Type of intervention	Including	No. of studies	Evidence
Societal recovery	Approaches aiming at personal goals, (social) skills training, occupational therapy	22	4 added value 11 promising results 7 no evidence yet
Personal recovery	Peer run, empowerment, confidence, hope, meaning	6	2 added value 4 promising results
Functional recovery	Cognitive remediation/training, cognitive adaptation	5	3 added value 2 no evidence yet
Lifestyle	Health promotion, exercise, healthy meals	13	7 promising results 6 no evidence yet
Spiritual and creative	Tai chi, music therapy, art therapy	7	3 added value 3 promising results 1 no evidence yet

Most of the included studies focused on societal recovery (*n* = 22), addressing psychiatric rehabilitation approaches, occupational therapy and skills training. Studies concerned with personal recovery (*n* = 6) focused on peer-run programs, illness management and recovery, and interventions aiming at increasing empowerment. Studies in the functional recovery category (*n* = 5) examined cognitive training or remediation. Those in the lifestyle category (*n* = 13) were aimed at a healthy lifestyle, (e.g. physical exercise and healthy eating). The last category, cultural and spiritual interventions (*n* = 7), looked at tai chi, music therapy and art therapy.

### Evaluation of Results of the Interventions

We evaluated the outcomes of all included studies (see Table 2 for a summary). Following is a description of the overall picture for each category.

**Table 2:** Results of qualitative synthesis

Authors	Design and study duration	Setting	Study population (N)	Intervention	Main outcomes	Main findings	Added value/promising first result/no evidence for effectiveness yet
Societal recovery							
Park and Han (2018)	Quasi-experimental pretest–posttest Duration: 5 weeks	Rehabilitation centers	People with chronic schizophrenia (n = 41)	CEP-S: Communication Enhancement Program	Communication skills Empathy Relationship skills Problem-solving skills	Increased communication skills and relationship skills	Promising first results
Beentjes et al. (2018)	Exploratory cluster RCT Duration: 12 months	Extensive inpatient and/or outpatient psychiatric treatment including case management at nine MHC institutes, including supported housing	People with SMI (N = 41)	e-IMR + IMR	Illness management, self-management, recovery, symptoms, quality of life, and general health	No significant results and low e-IMR use	No evidence for effectiveness yet
Sheridan et al. (2018)	Qualitative, written diary data Duration: 9 months	Mental health services including 28% supported accommodation	People with enduring mental illness (N = 34)	Volunteer partner group, supported socialisation programme to stimulate social/leisure activities	n/a	Positive findings on: involvement ‘normalising’ life, sense of connectedness, physical health, and facilitating engagement with culture, integrate socialising into identity, perceived social capacity	Promising first results
Bitter et al. (2017)	Cluster RCT Duration: 20 months	Sheltered/supported housing facilities	People suffering from SMI (N = 263) 71% inpatients	Comprehensive approach to rehabilitation (CARe) Methodology	Functioning Personal recovery Quality of life	Quality of life increased and amount of care needs decreased in both groups	No evidence for effectiveness yet
Loi et al. (2016)	Pre-post, non- randomized, study Duration: 6 weeks	Residential facility	Older adults suffering from SMI (N = 5)	Short educational training course on using the internet and touch screen	Social isolation Self esteem Internet use	No sign improvements or worsening in both outcomes	No evidence for effectiveness yet
Magliano et al. (2016)	Controlled non-randomized study Duration: 2 months	Residential facilities	People suffering from SMI (N = 114)	VADO Approach: Skills assessment and definition of goals (based on Falloon’s CBT and inspired by Boston (or choose-get-keep) approach)	Functioning	Positive result on functioning	Promising first results
Killaspy et al. (2015)	Cluster RCT Duration: 12 months	Inpatient rehabilitation units	People suffering from SMI (N = 344)	Staff training program designed to increase patients’ engagement in activities	The degree to which patients were engaged in activity over the previous week	No difference between the groups in engagement in activities	No evidence for effectiveness yet
Sanches et al. (2015)	Multi site RCT Duration: 12 months	FACT teams and supported and sheltered housing facilities	People suffering from SMI	Boston university approach to psychiatric rehabilitation (BPR; aka choose-get-keep)	Societal participation Patients’ experience of success Quality of life Recovery	Protocol	Results not known yet
Anthony et al. (2014)	Pre-post study Duration: 18 months	28 service programs	People suffering from SMI (N = 238) 49% sheltered facility	Residential and employment goal setting procedure in a choose-get-keep rehabilitation program	Employment status Residential status Earnings	Participants with residential goals improved sign on residential status and earnings; intervention completers improved on employment status – Participants with employment goals improved significant on employment status and earnings	Promising first results
Lindstrom et al. (2012)	Prospective pre-test, post-test, and follow up test Duration: 6 months	Supported or sheltered housing facilities	People suffering from SMI (N = 17) 82% inpatients	Home based occupational therapy intervention aiming at identifying, realising and sustaining meaningful daily occupations	Goal attainment Motor and process skills Social interaction Satisfaction with daily occupations ADL Psychiatric symptoms	Sign improvements on goal attainment, social interaction, and satisfaction with daily occupations, ADL and psychiatric symptoms	Promising first results
Ellison et al. (2011)	Pre-post design Duration: 12 months	State-wide implementation in several community facilities and supervised facilities	People suffering from SMI (N = 511 and 221) controls for the analysis of service use and costs (40% inpatients)	Intensive psychiatric rehabilitation based on choose-get-keep model	Role functioning on several domains Service use and service costs	A positive effect on residential status and earnings for completers	Promising first results
McMurran et al. (2011)	Pragmatic multi centre RCT Duration: 1.5 year	Community settings including residential or supported care settings	340 planned suffering from personality disorder	Psycho education combined with problem solving (PEPS) therapy	Social Functioning (SFQ)	Protocol	No results yet
Fagan-Pryor et al. (2009)	Retrospective outcome evaluation Duration: 3 years prior to- and 3 year post-implementation	Inpatient psychiatric facility	Male veterans suffering from SMI (N = 47)	Psychiatric rehabilitation and recovery based program based on choose-get-keep model with focus on housing	Discharge Community tenure Number of admissions	– Significant larger community tenure in discharged participants pre-post implementation	Promising first results
Levitt et al. (2009)	RCT Duration: 12 months	Supportive housing	104 persons with SMI	Illness management and recovery	Illness Management and Recovery Scales Psychosocial functioning Quality of life Symptoms	Significant difference in self-reported and clinician ratings of illness management, symptoms and psychosocial functioning of the quality of life scale	Added value
Pratt et al. (2008); Mueser et al. (2010)	RCT Duration: 3 years	Community residents,	Older adults (> + 50 years) suffering from SMI (N = 183) 50% inpatients	HOPES program: Social skills training and health management; 24 months	Psychosocial functioning Community functioning Self-efficacy Health	– Significant improvements in performance measures of social skills, psychosocial and community functioning, negative symptoms, and self-efficacy	Added value
Vandevooren et al. (2007)	Retrospective repeated measures design Duration: Prior to program: Annually over a 6-year period, before and after, 1 year follow up	Residential home	People suffering from SMI (N = 25)	Systematic rehabilitation approach based on choose-get-keep model	Community tenure Number of admissions Living situation	– Significant change in community tenure over 7 year period	Promising first results
Seo et al. (2007)	Quasi experimental design Duration: 2 months	Inpatient ward in psychiatric hospital	Persons diagnosed with schizophrenia (N = 74)	Social skills group training based on Liberman and Bellack modules	Social skills Self esteem Assertiveness skills Problem-solving skills Conversational skills	Differences in improvements of a number of social skills and self-esteem in favour of the intervention group	Promising first results
Pioli et al. (2006)	Partially randomized multi centric trial Duration: 12 months	Residential and day care centres	People diagnosed with schizophrenic disorder (N = 98) 33% living in sheltered facilities	VADO: Skills assessment and definition of goals	Social functioning Psychiatric symptoms	Significant improvement on psychiatric symptoms and social functioning	Promising first results
Rogers et al. (2006)	RCT Duration: 24 months	Intensive care receivers of State Department of Mental Health	Adults suffering from major mental illness (N = 135) 50% inpatients	Psychiatric vocational rehabilitation (PVR) using choose-get-keep model	Psychiatric symptoms Quality of life Self esteem Vocational & educational status	No sign differences over time in employment status, symptoms, quality of life or self-esteem	No evidence for effectiveness yet
Oka et al. (2004)	Retrospective study Duration: Minimal 3 yrs. follow up	Previously long term hospitalized persons, recently discharged and living independently or in a residential home	Persons diagnosed with schizophrenia (N = 52)	Hybrid occupational therapy and supported employment	Hospitalization Community tenure Social functioning	Social functioning improved significantly greater after supported employment was started Mean number of hospitalization decreased Community tenure increased significantly	Promising first results
Anzai et al. (2002)	RCT Duration: 1 year	Inpatient facility	Persons diagnosed with schizophrenia (N = 32)	Illness self-management skills training program based on the community re-entry module of Liberman et al.	Psychotic symptoms Knowledge and skills Rehabilitation skills	Significant improvement in knowledge and (rehabilitation) skills in the intervention group Patients in the intervention group spent significantly more time in community in comparison to the control group	Added value
Tsang and Pearson (2001)	Cluster randomized pilot test Duration: 3 months	Community-based staffed residential facilities	Persons diagnosed with schizophrenia (N = 97)	Social skills training in the context of vocational rehabilitation	Work related social skills, self-perceived Social skills in role play exercise Job motivation checklist Vocational outcome and adjustment	Work related social skills; self-perceived and measured with role play were both significantly higher in the two training groups Training group with follow up support most successful in job search	Added value
Personal recovery							
Nowak et al. (2018)	Pre-post evaluation Duration: 6 weeks	Clinics	People diagnosed with schizophrenia (N = 46)	Recovery-oriented cognitive behavioral workshop	Recovery Psychosocial functioning	No significant change over time in total recovery Improvement regarding confidence and hope, feeling less dominated by symptoms, psychosocial functioning and psychopathology	Promising first results
Boevink et al. (2016)	RCT Duration: 24 months	2 community treatment teams and 2 sheltered housing organisations	Persons suffering from severe mental illness (N = 163) 28% inpatients	User run recovery programme TREE	Empowerment Mental health confidence Loneliness	Sign more mental health confidence Less care needs Less self-reported symptoms Less likelihood of institutional residence	Added value
Mancini et al. (2013)	Quasi-experimental design Duration: 6 months	Psychiatric hospitals	People suffering from SMI (N = 110)	Pro-recovery; a 14-week consumer developed approach including structured group-sessions	Pro-recovery Evaluation Instrument: social satisfactions; quality of life, well-being, recovery	Significant effect on consumer’s perception of the recovery attitudes of staff	Promising first results
Park and Sung (2013)	Repeated-measure design with matched controls Duration: 10 weeks	Psychiatric hospitals	Persons diagnosed with schizophrenia (N = 46)	The empowerment program for schizophrenic patients: A nursing intervention focusing on patients’ strength and hopes of recovery	Helplessness Recovery (patient report and nurse report)	Significant effect on helplessness and recovery	Added value
Willemse et al. (2009)	Pilot evaluation Duration: 12 weeks	Long stay ward of three psychiatric hospitals and one sheltered housing	Older people (mean age: 67) (N = 36)	Searching for meaning in life-program	The Philadelphia geriatric center morale Quality of life	Significant increase in life satisfaction	Promising first results
Randal et al. (2003)	Matched control evaluation study Duration: depending on individual trajectories	Inpatient rehabilitation unit	9 people with treatment resistant schizophrenia or schizoaffective disorder	Individual, flexible, recovery-focused multimodal therapy (21 months)	Positive and negative symptoms, rehabilitation	Reduction in positive symptoms, negative symptoms, and in general psychopathology symptoms. General behavior scores on the Rehabilitation Evaluation of Hall and Baker were clinically improved	Promising first results
Functional recovery							
Schutt et al. (2017)	Pre-post pilot study Duration: 2 months	Group home	6 residents	Cognitive remediation	Neurocognitive performance	No significant gains in cognitive performance	No evidence for effectiveness yet
Stiekema et al. (2015)	Cluster RCT Duration: 24 months	Long stay departments of 3 institutions	100 planned	Cognitive adaptation training of nurses and specialists	Executive functioning Cognitive strengths and weakness Everyday functioning Quality of life Empowerment	Protocol	Results not known yet
Sánchez et al. (2013)	RCT Duration: 3 months	Psychiatric hospital	Persons diagnosed with schizophrenia (N = 84)	REHACOP, integrative cognitive remediation program that taps all basic cognitive functions	Neuro-cognition Clinical symptoms Functioning	Significant effect on neuro-cognition, negative symptoms, disorganization, and emotional distress	Added value
Lindenmayer et al. (2012)	RCT Duration: 3 months		Persons diagnosed with schizophrenia (N = 59) (93% inpatients)	Cognitive remediation (CR) + social cognitive intervention	Social cognition and neurocognitive functions, psychopathology and social functions	Combined CR with emotion perception remediation produced greater improvements in emotion recognition, emotion discrimination, social functioning, and neurocognition compared with CR alone	Added value
Medalia et al. (2001)	RCT Duration: 5–6 weeks	Inpatient psychiatric centre	Persons with schizophrenia (N = 54)	Remediation of cognitive problem solving skills	Independent community living Verbal knowledge, judgement, and problem solving Verbal memory and narrative recall	For independent living change scores, a significant between-group difference was found	Added value
Healthy lifestyle							
Looijmans et al. (2019)	Multi-site randomized controlled pragmatic trial Duration 12 months	Flexible Assertive Community Treatment (F- ACT) teams and sheltered living teams	SMI patients (N = 140)	Multimodal lifestyle approach, including a web-based tool to improve patients’ cardiometabolic health	Primary: differences in waist circumstance at 6 and 12 months Secondary: BMI and metabolic syndrome Zscore	No statistical significant differences found on the p and s outcomes Readiness to change dietary behavior improved	No evidence for effectiveness yet
Sweeney et al. (2019)	RCT and cost effectiveness evaluation Duration 8 months	Residential and non-residential community mental health services	Smokers with SMI (N = 382)	Quitlink utilizing the existing mental health peer workforce to link SSMI to a tailored smoking quitline service	Continuous abstinence Secondary: 7-day abstinence, increased quit attempts, and reductions in cigarettes per day, cravings and withdrawal, mental health symptoms and other substance use, and improvements in quality of life	Protocol	No evidence for effectiveness yet
Ringen et al. (2018)	Prospective naturalistic intervention study Duration: 7 months	University hospital and a private inpatient psychiatric care facility	Long term inpatients (N = 83)	Motivational interventions, psychical activity and establishment of a basic infrastructure regarding activity and diet	Psychical activity, motivation, self-esteem, life satisfaction, functioning, symptoms	No increase of physical activity level. Triglyceride levels and numbers of smokers were significantly reduced and a significant decrease in symptom levels was observed	No evidence for effectiveness yet
O’Hara et al. (2017)	Structured interviews and qualitative data: two focus groups and field notes Duration: 12 weeks	Supportive housing	People with SMI	Peer based group Lifestyle balance	Feasibility, acceptability, adaptations	Participants attended on average 8/12 sessions Perceived it as helpful and satisfactory	Promising first results
Looijmans et al. (2017)	Cluster RCT Duration: 12 months	Residential and long-term teams of 2 mental health care organizations	People suffering from severe mental illness (N = 371)	Lifestyle intervention focusing on cardio metabolic health	Waist circumference Body mass index Metabolic syndrome z-score	Waist circumference decreased 1.51 cm in the intervention group versus control group after 3 months and metabolic syndrome z-score decreased 0.22. After 12 months, the decrease in waist circumference was no longer significant	Promising first results
Hjorth et al. (2016)	Cluster RCT Duration: 12 months	Longterm psychiatric treatment facilities	Staff members serving as role models for severely and chronically mental ill patients (N = 174)	Health promotion intervention for staff as role modelling for patients	Waist circumference BMI Weight Lung PEEP Blood pressure Physical fitness Tobacco and alcohol consumption Quality of life	No effects found on client level There was a relation in: Staff and patient change in quality of life	No evidence for effectiveness yet
Hutchison et al. (2016)	Pre-post study Duration: 12 months	Long term residential mental health care facility	Persons suffering from with severe mental illness (N = 43)	In SHAPE program, a health promotion program aiming at physical activity and healthy diet, using assessment, fitness plan, weekly meetings education, incentives, and group motivational celebrations	Physical activity Physical health Recovery Severity of depression Self-perceived ability to implement health-promoting behaviors Hopefulness	100% expressed a nutrition and exercise goal, and weekly logs were filled in by the majority Physical activity, health has increased Recovery and depression improved significantly Self-perceived ability improved for wellbeing and exercise	Promising first results
Gill et al. (2016)	Pilot: Single group pre-post design Duration: 8 weeks	Supported housing programs and ACT program	Adults with serious mental illnesses (N = 77)	Wellness for life inter-professional health promotion intervention Including: Exercise, nutritional counselling, health literacy education, and peer wellness coaching	Blood pressure Blood glucose Waist circumference Body weight Physical strength and flexibility BMI Readiness to change Health status	Average blood pressure and waist circumference decreased Strength and flexibility improved Readiness for diet and exercise improved	Promising first results
Loh et al. (2016)	Pilot RCT Duration: 3 months	Long stay ward	Patients diagnosed with schizophrenia (N = 104)	Structured walking intervention	Health related quality of life	Positive effect on quality of life, wellbeing and psychiatric symptoms	Promising first results
Cabassa et al. (2015)	RCT Duration: 18 months	Supportive housing	300 planned	Peer-led healthy lifestyle program	Weight Quality of life Recovery	Protocol	No results yet
Oertel-Knochel et al. (2014)	Matched pre-post design Duration: 1 week before and 1 week after the intervention		Long-term patients suffering from a major depression or schizophrenia (N = 51)	Exercise group: Cognitive training + aerobic exercise Relaxation group: Cognitive training + relaxation 12 sessions in for weeks	Cognitive performance Symptoms Wellbeing	Increase in cognitive performance in the domains visual learning, working memory and speed of processing, a decrease in state anxiety and an increase in subjective quality of life between pre- and post-testing	Promising first results
Verhaeghe et al. (2013)	Cluster preference RCT Duration: 6 months	Sheltered housing organisations	Adults with mental disorders (N = 324)	Health promotion program aiming at physical activity and healthy eating	Body weight BMI Waist circumference Fat mass Health-related quality of life Psychiatric symptom severity	Significant results on body weight, BMI, waist circumference, fat mass, however disappeared during follow up except for fat mass	Promising first results
Forsberg et al. (2010)	Cluster RCT Duration: 12 months	8 Supported housing facilities and 2 housing support programmes	Persons with severe mental illness (N = 41)	12 month Lifestyle intervention program	Quality of life Functioning Psychiatric symptoms	No difference found between the study groups	No evidence for effectiveness yet
Spiritual and creative							
Berry et al. (2016)	Cluster RCT Duration: 6 months	Psychiatric rehabilitation wards	Patients with complex mental health needs (N = 51 patients and 85 staff)	24 one-hour sessions focussing on staff-patients relationships per ward over 6 months	Staff and patient relationships Staff wellbeing Patient functioning	Significant less depersonalization in staff Less feeling of criticism by patients and improvement of ward organization and relationships by patients	Added value
Ho et al. (2014)	3-arm RCT Duration: 24 weeks	Residential rehabilitation complex	Patients diagnosed with schizophrenia (N = 153)	Tai chi	Symptom management Motor coordination Memory Daily living function Stress levels	Protocol	No results yet
Gold et al. (2013)	Pragmatic parallel trial Duration: 9 months	Specialised mental health care settings	Adults with severe mental disorders (N = 144)	3 months biweekly individual resource-oriented music therapy	Negative symptoms General symptoms Motivation for change Self-efficacy Self-esteem Social relationships	Effect on negative symptoms, functioning, clinical global impressions, social avoidance through music, and vitality	Added value
Kwon et al. (2013)	Quasi-experimental pretest–posttest design Duration: 7 weeks	Mental health rehabilitation complex	Adults with severe mental disorders (N = 55)	7 week group music therapy	Brain wave, cognitive function, behavior	Effect on alpha waves revealing that the participants in the music therapy may have experienced more joyful emotions throughout the sessions. The experimental group also showed improved cognitive function and positive behavior (social competence, social interest & personal neatness) while their negative behaviors was significantly less	Promising first results
Ho et al. (2012)	Pilot RCT Duration: 12 weeks	Mental health rehabilitation complex	Patients with chronic schizophrenia (N = 30)	Tai chi (6 weeks)	Movement coordination Negative symptoms Disability	Effect on movement coordination and interpersonal functioning. Fewer disruptions to life activities at 6 weeks after the intervention	Promising first results
Gelkopf et al. (2006)	Cluster randomized trial Duration: 3 months	Psychiatric hospital	Patients with chronic schizophrenia (N = 29)	Humorous movies Daily for 3 months	Positive and negative symptoms Anxiety Depression Anger Social functioning Treatment insight Therapeutic alliance	Significant larger difference over time in reduction of negative symptoms, depression and anxiety than in control group The intervention group showed a significant larger improvement in time than the control group on the social functioning scale	Added value
Hayashi et al. (2002)	Non randomized, controlled study Duration: 4 months	Long stay wards of mental health care institute	Female patients with chronic psychoses (N = 66)	Group musical therapy Including, listening to and making music and group communication about it	Psychotic symptoms Objective quality of life Subjective musical experiences Ward activity and—adjustment	A significant advantage was found of the intervention for psychotic symptoms, quality of life, musical experience, and ward activity over time during the intervention Effects did not last at follow up	Promising first results

### Societal Recovery

This category contains the greatest number of studies (*n* = 22). These studies focussed on diverse interventions. Nine evaluated interventions aimed at general goal achievement, seven at achieving specific and/or disability management and two at vocational rehabilitation. One study concerned a staff-training program designed to increase patients’ engagement.

Of the nine studies that evaluated interventions aimed at goal attainment, seven interventions were totally or partly based on the ‘choose-get-keep’ model (Anthony et al. 2014; Ellison et al. 2011; Fagan-Pryor et al. 2009; Magliano et al. 2016; Pioli et al. 2006; Sanches et al. 2015; Vandevooren et al. 2007). Three of the goal attainment studies were RCTs, and four were uncontrolled/pre-post design. Five of these studies showed (small) positive results (Ellison et al. 2011; Fagan-Pryor et al. 2009; Magliano et al. 2016; Pioli et al. 2006; Vandevooren et al. 2007), among others, concerning functioning and residential status. Bitter et al. (2017) evaluated, by means of a cluster randomized trial, CARe: A rehabilitation approach based on the strengths model and personal recovery in teams of supported accommodation, but did not find any differences in outcomes between the clients of trained and untrained teams.

Of the studies on interventions concerning skills and illness/disability management, two RCT studies evaluated the illness management and recovery (IMR) approach (Beentjes et al. 2018; Levitt et al. 2009). The Levitt study reported significant improvements in illness management, symptoms and psychosocial functioning, while the Beentjes’ e-IMR study did not due to low implementation rates. Lindström et al. (2012) conducted a study on a home-based occupational therapy intervention aiming at daily occupations including remediation and compensatory strategies. The authors observed positive significant results on most outcomes (goal attainment, social interaction, satisfaction with daily occupations, activities of daily living (ADL) and psychiatric symptoms). Anzai et al. (2002) examined an RCT on an training program for illness management skills based on Liberman’s community re-entry module, resulting in positive effects including knowledge and skills and community participation. In a small, pre-post study on a short educational training course on using the internet and touch screen, no effects were found on social isolation, self-esteem and internet use (Loi et al. 2016). Three studies (Park and Han 2018; Seo et al. 2007; Tsang and Pearson 2001) examined societal recovery explicitly focussed on social skills. Tsang and Pearson (2001) evaluated social skills training in the context of vocational rehabilitation. This cluster randomized pilot found positive results for work-related social skills, motivation to seek employment and success in job search. Seo et al. (2007) conducted a quasi-experimental study on social skills group training that included conservational and assertiveness skills based on the Liberman modules. The results showed a difference in improvement of social skills and self-esteem in favour of the intervention group. Park and Han (2018) studied with a quasi-experimental design a 5-week communication program based on communication theory of Walsh and existing of ten sessions. They found improved communication, and relational skills, but no improvement in problem solving, though used an alpha of 0.70.

Two studies evaluated interventions aimed at vocational rehabilitation. Oka et al. (2004) evaluated a hybrid occupational therapy and supported employment intervention by means of a retrospective study. Positive results were achieved concerning social functioning and hospitalisation. Rogers et al. (2006) evaluated the choose-get-keep approach in a vocational context compared with enhanced state vocational rehabilitation and found no differences between the groups. A positive effect on vocational status was found for both interventions, indicating that a rehabilitation approach aiming at work can be effective for this group.

Finally, the remaining studies were concerned with client engagement in activities (Killaspy et al. 2015; Sheridan et al. 2018) and psychoeducation based on cognitive behavioral therapy (McMurran et al. 2011). Killaspy et al. (2015) evaluated a staff-training program designed to increase patients’ engagement in activities. In this cluster-randomized trial, no differences were found between the study groups in engagement in activities. Sheridan et al. (2018) studied the effects of a supported socialisation volunteer partner group to stimulate social and leisure activities. In their qualitative thematic analyses of diary data they found indications for positive effects on involvement in normalising life, connectedness, physical health, social capacity and culture engagement. McMurran et al. (2011) published on a protocol to evaluate a 12-session group intervention aimed at problem solving.

### Personal Recovery

The six studies in this category evaluated interventions aimed at personal recovery (including outcomes on empowerment, hope, confidence, and quality of life or comparable). All studies showed added value or promising first results. Of these studies, one was an RCT and five were semi-controlled or pre-post designs. Two studies were peer-run interventions. One of these peer-run interventions examined confidence and care needs (Boevink et al. 2016) and the other on consumers’ perception of the recovery attitudes on the staff (Mancini et al. 2013).

One study focussed especially on elderly patients and showed a small but positive result concerning life satisfaction (Willemse et al. 2009). Park and Sung (2013) reported results of a study on a 6-week, recovery-oriented nursing intervention. This study also showed positive results on helplessness and recovery, but due to the non-controlled design, these results need further confirmation in replication studies. There were two studies on therapies to enhance personal recovery (Nowak et al. 2018; Randal et al. 2003). Randal et al. (2003) conducted a small, matched-control evaluation study on individual recovery-focused multimodal therapy. Following Evans’ design hierarchy, the results can be interpreted as promising with outcomes showing significantly more improvement of positive and negative symptoms and a decrease of deviant behavior, e.g. verbal aggression and violence. Nowak et al. did a pre-post study on a recovery-oriented cognitive behavioral workshop of 6 weeks. They found no significant change in total recovery, but they did find significant improvements in sub scales including confidence, hope and psycho social functioning.

### Functional Recovery

This category included five studies evaluating interventions focused on improvement of cognitive and executive functions. Four were RCTs, and one had a pre-post design. A study on an integrative program that focused on all basic cognitive functions showed positive results concerning vocational outcomes, family contact and social competence (Sánchez et al. 2013). Lindenmayer et al. (2012) conducted an RCT on an intervention that combined cognitive remediation with social cognition training. The combined intervention resulted in greater improvements in emotion recognition, emotion discrimination, social functioning and neuro-cognition compared with cognitive remediation alone. Another study resulting in interesting results was a cognitive remediation intervention focusing on problem solving skills (Medalia et al. 2001). This study found a significant difference for independent living. Schutt et al. (2017) executed a small pre-post study on a cognitive remediation intervention, but did not find relevant outcomes. Stiekema et al. (2015) published on their protocol to evaluate a cognitive adaptation training (CAT).

### Healthy Lifestyle

We found thirteen studies focusing on lifestyle interventions; all were published after 2010. Seven were RCTs, five were semi-controlled or pre-post studies and one was qualitative. Seven of these studies showed promising first results, four did not show evidence and two were protocol papers. Loh et al. (2016) executed a (pilot) RCT on a structured walking intervention. In this study, the participants of the control group scored slightly better on quality of life, psychiatric symptoms, physical role limitations and physical functioning after 3 months. Hjorth et al. (2016) evaluated an intervention program for improving physical health in staff and its impact on patient’s health. The intervention had a positive effect on the waist circumference and blood pressure for the staff, and there was a statistically significant association between the staff change in each facility and the patients’ change in health parameters.

Looijmans et al. (2017) conducted a cluster RCT on lifestyle intervention that focused on cardio metabolic health. This intervention led to positive results after 3 months on waist circumstance and metabolic syndrome. The same research group studied the use of a web-based tool (Looijmans et al. 2019) in FACT teams and sheltered living teams. Findings indicate no significant improvements on the primary and secondary outcomes and an improvement on the readiness to change. Oertel-Knöchel et al. (2014) conducted a combined cognitive–aerobic/relaxation intervention showing that physical exercise is a valuable addition to cognitive training. Verhaeghe et al. (2013), Cabassa et al. (2015), Forsberg et al. (2010) and O’Hara et al. (2017) also all studied a lifestyle program. Verhaeghe et al. conducted a cluster RCT on a comprehensive lifestyle intervention (psycho-education, supervised exercise and individual support) in sheltered housing services. Although initially small positive results were achieved on weight, body mass index (BMI) and waist circumstances, these results almost all disappeared during follow-up. No differences were found regarding secondary outcomes (i.e., symptoms and quality of life). Cabassa et al. published a study protocol. Forsberg et al. did not find support for the added value. O’Hara et al. studied the results of a peer based group and did this qualitatively, using focus groups and field notes, and added this with structured interviews. The results indicate participants attended on average three quarter of the sessions and perceived them as helpful and satisfactory. Ringen et al. (2018), Hutchison et al. (2016) and Gill et al. (2016) all executed pre-post evaluations on a promotion / motivational program, of which the first did not show improvements and the latter two resulted in positive results on physical activity and physical health. Sweeney and Baker, finally, published two protocol papers on an intervention in which existing peer workers tailor clients to appropriate smoking quitline service (Sweeney et al. 2019).

### Spiritual and Creative Therapy

This category contained seven studies. Two studies (one protocol) evaluated Tai chi (Ho et al. 2012, 2014) of which a pilot RCT showed promising results concerning movement and interpersonal functioning. Three studies (Gold et al. 2013; Hayashi et al. 2002; Kwon et al. 2013) evaluated a form of music therapy. In all studies, positive results were achieved concerning amount others: Negative symptoms (Gold et al. 2013), cognitive function (Hayashi et al. 2002), positive behavior (Kwon et al. 2013), and quality of life (Hayashi et al. 2002). These positive results, however, did not last through the last follow-up.

One study in this category evaluated the effect of watching humorous movies. Watching these movies regularly for 3 months appeared to have a small positive effect on negative symptoms, depression and anxiety, and social competence (Gelkopf et al. 2006). The seventh study was a cluster trial on a ward intervention to improve patient-staff relationships and wellbeing leading to significant differences in depersonalization in staff and criticism experienced by clients (Berry et al. 2016).

## Discussion

With this study, we aimed to achieve insight into which psychosocial interventions are available to support recovery in other dimensions than the clinical one and evaluated in people with SMI who live in supported accommodations. Additionally, we explored what scientific knowledge is available about the outcomes of these interventions. We found 53 studies with different types of interventions aiming at several non-clinical dimensions of recovery. Almost a quarter (22.6%) of these interventions showed added value and almost half of them (47.2%) first promising results. This is a hopeful result that shows that improvement on recovery is possible, even for people with SMI living in supported accommodations which are, as shown in the introduction, often dealing with long-term and complex needs. The articles included in this study provide knowledge concerning the current use of psychosocial interventions in supported accommodations and give us new insights in the opportunities for implementation, further development and evaluation of interventions.

These findings indicate that there have been some practice and research attention for the other dimensions of recovery for the group of people who need supported accommodation in the last 20 years. Interventions aimed at societal recovery have the longest tradition in general mental healthcare, which is reflected in the larger number of papers found for the group living in supported accommodations and their publication date as well. Of these, most interventions were based on the Boston choose-get-keep rehabilitation approach which showed inconsistent results, some no added value, some promising results. Further study needs to bring answers to when, for whom and why these interventions do or do not work. Realist evaluations are the most suiting design for this (Wong et al. 2016). Interventions showing the most consistent added value included IMR and social and self-management skills trainings and are therefore relevant to follow and replicate.

Additionally, we found small amounts of papers concerning the two other known recovery dimensions: personal and functional recovery. Developments which are relevant to follow and replicate if we truly want the whole group with SMI to profit from the paradigm shift in mental health care towards a broader definition of recovery in which more recognition exists for the personal experience of people with mental illnesses (Leamy et al. 2011). On personal recovery, markedly, all six interventions found had added value or promising results. Noteworthy are the two interventions with added value: the TREE peer-to-peer intervention and the empowerment program provided by nurses. Of the five functional recovery interventions, three showed added value, which all included cognitive remediation interventions. Cognitive adaptation training have not been studied frequently, but is one to follow: it is in concept easy to implement and if effective, a large contribution to independent functioning can be expected. Interventions on the functional recovery dimension are especially relevant when considering that cognitive dysfunction and related negative symptoms can be strong obstructing factors in the life of people with severe mental health problems (Quee et al. 2014; Stiekema et al. 2016).

Additional to the known recovery dimensions, we found a relatively large number of studies on healthy lifestyle (13) and on the spiritual and creative domain (7). Healthy lifestyle is a relevant life area as a substantial number of people suffering from a severe mental illness are affected by comorbid medical conditions which influence their life expectancy, quality of life and recovery on other dimensions (Scott and Happell 2011). No interventions showed added value, but half of them were promising. Noteworthy is that most of the health promotion interventions, all including exercise and some a healthy diet as well, showed promising results. Interesting was the structured walking intervention showing promising results, which seems an easy to implement intervention with large impact. Five interventions: the peer led, smoking, web-based, and the two health promotion/motivational interventions by staff did not show added value. The results indicate that concrete lifestyle programs might add more to the results.

In the spiritual and creative intervention category three music therapies were studied of which, noteworthy, one showed added value and two promising results. Tai chi was twice studied as intervention: one showing no results and one promising results. Markedly, humorous movie watching as intervention showed added value. This finding relates to current insights: cultural interventions have high potential for health gains as was recently underlined in a scoping review of the WHO (Fancourt and Finn 2019).

This broader scope and promising results are hopeful developments especially as people with severe mental illness experience several unmet needs (Bitter et al. 2016; de Heer-Wunderink 2012; Wiersma 2006). However, compared with the ambulatory treated people with mental illness, the number of studies we found on recovery can still be considered relatively low (van Weeghel et al. 2019b). This is not surprising because since the start of deinstitutionalisation in the second half of the twentieth century, the focus of practice, research and policy increasingly shifted towards the development of ambulant and community-oriented services (Burns and Firn 2017). Although this was an important development in mental health care, which led to the increasing opportunity for people with SMI to participate in society, the risk exists that a knowledge gap emerges concerning the group in need supported accommodation (McPherson et al. 2018). It is therefore important that more studies focus on this group to gain more insight in what these people need in their recovery and to develop interventions that match their needs.

### Strengths and Weaknesses of the Study

This study has several strengths and limitations. A strength was the broad scope. Our aim was to provide an impression of psychosocial interventions that exist for people with SMI who need supported accommodation and to provide first insights into what is known about the effectiveness of these interventions. Therefore, we used a broad search strategy and included a variety of interventions aiming at a broad range of outcomes and executed in different settings and (international) contexts, and included all types of study designs. When developments in the recovery field are a bit further along, a quantitative synthesis would add to our knowledge. At this point the number of studies in the supported accommodation field is too small yet to perform a quantitative review (Chilvers et al. 2006; McPherson et al. 2018). A point of attention is that we used information provided in the included articles only, which sometimes was somewhat poor, for example not all papers published effect sizes. So, it might be that the quality of some papers is displayed more positively if it was based on the p values only. Another note is that when performing a review, a selection of specific search terms is chosen. There is always a risk that not all relevant papers end up in the results due to word use in titles, abstracts and key words. When reading this and other reviews, this should be kept in mind. Nevertheless, this study provides a broad overview of interventions on several dimensions of recovery besides the clinical one that can give supported accommodation an impression of interventions that may be relevant and sufficient to implement, and bring the recovery forward, even in people that cope with severe, and therefore often complex and long-term, mental illnesses.

### Suggestions for Development of Practice and Research

Research specifically focussing on the recovery dimensions besides clinical recovery of people with severe mental illness who live in supported accommodations remains limited but seems to be in development. We also can conclude that a broader vision towards recovery in these settings has gained attention and that, regarding all other dimensions of recovery, hopeful results have been achieved so far.

Four challenges can be appointed concerning the practice and research of interventions for people with severe mental illness who live in supported accommodations. The first challenge is the further development and professionalization of recovery-oriented care and support offered for this specific group of people. Effective and promising interventions should be developed and made available for all people with severe mental illness, despite their place in the care landscape (Couwenbergh and van Weeghel 2014).

The second challenge is to accompany developments in practice with research to gain more insight into what works, for whom and what does not, so that the provided care can be more personalized. Specific knowledge is needed concerning the group of people who are in need of supported accommodation. For example, we were surprised that for some well-known recovery interventions, for example, the wellness recovery action plan (WRAP) (Fukui et al. 2011) or narrative enhancement and cognitive therapy (NECT) (Fukui et al. 2011), no studies were found explicitly focussing on people living in supported accommodation. Here may lay a chance for further development, as it is worthwhile to study interventions that have proved themselves in ambulant contexts to see if they also can help clients with more complex and supported living needs.

The third challenge is the integration of different approaches towards recovery. In several countries, different forms of support are fragmentized (Boevink et al. 2016). For example, in the Netherlands a separation exists between clinical mental health care services and supported accommodation services. The insight is growing that integration of different aspects of recovery may lead to better outcomes (Corrigan et al. 2012). This might lead to improvement of recovery orientation of the care for people living in supported accommodation. Altogether, it is recommended that supported accommodation services reconsider their scope and position in the care landscape and consider broadening and strengthening their recovery-oriented services, as well as stronger collaborations between stakeholders including mental health treatment providers, supported housing organisations and local organizations for community support.

The fourth challenge is the professionals’ interest, knowledge and implementation skills to adapt and use state-of-the-art interventions. Working evidence based asks for an innovative mind set as well as time and support in keeping up-to-date and using new interventions that were proven effective in research.
